# A Dog with Pseudo-Addison Disease Associated with *Trichuris vulpis* Infection

**DOI:** 10.1155/2011/682039

**Published:** 2011-06-08

**Authors:** Luigi Venco, Valentina Valenti, Marco Genchi, Giulio Grandi

**Affiliations:** ^1^Veterinary Hospital “Città di Pavia”, Viale Cremona 179, 27100 Pavia, Italy; ^2^Dipartimento di Patologia Animale, Igiene e Sanità Pubblica Veterinaria, University of Milan, Veterinary School, Via Celoria 10, 20133 Milano, Italy; ^3^Dipartimento di Salute Animale, University of Parma, Veterinary School, Via del Taglio 10, 43100 Parma, Italy

## Abstract

A female Rottweiler dog was presented with a history of intermittent vomiting and diarrhoea, dysorexia, weakness, and weight loss. Haemocytometry and biochemistry values were within normal ranges except for electrolyte analyses, that demonstrated hyponatremia and hyperkalemia with a decreased sodium/potassium ratio. A diagnosis of hypoadrenocorticism was suspected. Basal and post-ACTH stimulation cortisolemia were within the normal values. Electrocardiography was normal, and thoracic radiography showed no significant modifications. On abdominal ultrasonography, adrenal glands appeared normal, while the bowel was distended, and several thin linear hyperechoic objects floating in the lumen were observed. Two adult female whipworms (*Trichuris vulpis*) were collected following bowel irrigation. Anthelmintic treatment against the parasite was curative.

## 1. Introduction

*Trichuris vulpis*, commonly known as whipworm, is characterized by a direct life cycle and by the extremely resistant lemon-shaped eggs that can remain infective in the environment for several years. Usually, *T. vulpis* infections are asymptomatic, but the presence of high worm burdens in the large intestine may cause the occurrence of haemorrhagic colitis due to the continuous stimulation and damage to the mucosa, where the head of the worm is embedded and moves in search of blood and fluid. Electrolyte imbalance can be associated with helminth infection; however, *T. vulpis* seems to be the only nematode associated with pseudo-Addison disease, as the present case seems to demonstrate.

## 2. Case History

An 8-year-old, spayed female Rottweiler mixed breed dog was presented to our hospital with several weeks history of intermittent vomiting and diarrhoea (watery faeces with mucus and increased stool frequency) together with dysorexia, weakness, and weight loss. 

**Figure 1:**
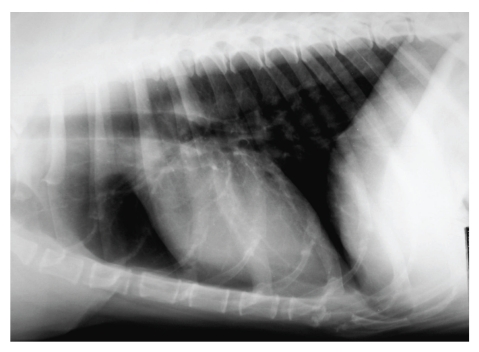
Thoracic radiograph laterolateral view showing the reduction of the cardiac silhouette (vertebral heart scale system score: 8.2) and pulmonary vascular hypoperfusion that both suggest reduction of plasmatic volume.

**Figure 2:**
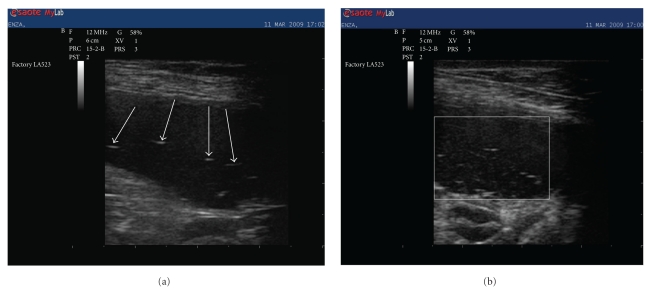
Abdominal ultrasonography (linear probe 10 MHz). In different views, the bowel appears distended by fluid with several thin linear hyperechoic objects (arrows (a)) (white square (b)) floating within the lumen.

On physical examination, the patient was weak, but alert. Heart rate, respiratory rate, and temperature were within reference ranges. Mucous membranes appeared tacky and pale. The dog was slightly dehydrated, thin (body condition score: 2.5/5, 33 kg), had mild muscle wasting, and generally poor body condition. Haemocytometry and biochemistry showed normal values except for electrolyte analyses that demonstrated hyponatremia (sodium 132 mmol/L; reference interval: 140 to 155 mmol/L), hyperkalemia (potassium 5.7 mmol/L; reference interval: 3.8 to 5.2 mmol/L), with a decreased sodium/potassium (Na : K) ratio (23: reference interval 27 to 40), all suggestive of hypoadrenocorticism [1]. A direct fresh smear faecal examination yielded a negative result for intestinal nematodes. Electrolyte abnormalities were, therefore, attributed to a possible hypoadrenocorticism and evaluation of basal and post-ACTH stimulation cortisolemia was performed. Electrocardiography, carried out while awaiting ACTH stimulation results, did not show any abnormality. Thoracic radiograph showed a small cardiac silhouette with a vertebral heart scale system score of 8.2 (reference values 8.5–10.6) [2] and reduced pulmonary perfusion (Figure 1). Abdominal ultrasonography was also carried out. Adrenal glands appeared normal both for dimension and morphology, while the bowel was distended by fluid, and several thin linear hyperechoic echoes floating within the bowel lumen were observed (Figures 2(a) and 2(b)). A bowel irrigation with warm saline solution was performed, and two adult female whipworms (*Trichuris vulpis*) were collected. Centrifugation flotation faecal analysis showed a few whipworm eggs with the characteristic “double plugs”, while the results of basal and post-ACTH stimulation cortisolemia were within the normal values.

The dog was treated with oral administration of milbemycine oxime (0.70 mg/kg bw; Interceptor Novartis AH) and i.v. administration of 800 mL of saline solution (Na^+^Cl^−^ 0,9%) and discharged.

Three day later, the dog appeared clinically improved, and electrolyte analyses were completely normal. One month later, the dog was rechecked and clinical hematological exams were within the normal range; faecal analysis was negative, and body condition was clearly improved (body condition score 3/5, 37 kg). Oral milbemycine oxime at the same dose as above was prescribed once a month for an entire year in order to avoid whipworm reinfection.

## 3. Discussion

Although uncommon, as general rule intestinal nematode infections should be considered as a possible cause of electrolyte imbalance [3]. To the authors' knowledge, however, only *T. vulpis* infections are reported as causing hyponatremia and hyperkalemia [4–7]. Although this kind of syndrome (pseudo-Addison disease) due to *T. vulpis* infection has been reported, its pathogenesis is not well understood. The symptoms mimic those of Addison's disease with waxing and waning weakness. Severe electrolyte disturbance ultimately creates dehydration. The syndrome mimics Addison's disease in every way except that testing for Addison's disease is negative and deworming yields a complete recovery [4–7]. In this case, the first diagnostic suspicion was hyponatremia as a consequence of the gastrointestinal losses, exacerbated by continued drinking and nonosmotic stimulation of antidiuretic hormone (ADH) release in response to volume depletion and hyperkalemia related to metabolic acidosis and decreased urinary excretion of potassium caused by reduced distal renal tubular flow rate [4, 6]. To note that this seems to happen only in the case of *T. vulpis* infection and not in other parasite infections that cause severe diarrhoea such as *Giardia intestinalis* or severe *Ancylostoma caninum* infections. Previous studies have shown that *T. vulpis* is able to induce transmural ileocolitis with severe lesions to the intestine wall and local cellular inflammation and oedema during the prepatent period of infection and fibrosis and mixed cellular inflammation of the terminal ileum, cecum, and proximal colon in response to deep penetration of adult worms, all of which could be responsible for electrolyte imbalance [8, 9]. 

This case report indicates the need to perform accurate faecal examination even when the dog's condition does not appear related to a parasitic infection and that dogs should be periodically monitored for helminth infection. In fact, the negative results of the fresh smear faecal examination confirmed the low sensitivity of this technique: 92.7% of whipworm false negatives when comparing direct smear to centrifugation [10].

This case report also suggests that whipworms in dogs, particularly in those cases where the bowel is distended by fluid allowing thorough examination, may be detected by ultrasonography as linear floating echoes, as previously demonstrated in humans [11].
